# Search for MHC/TCR-Like Systems in Living Organisms

**DOI:** 10.3389/fimmu.2021.635521

**Published:** 2021-05-04

**Authors:** Julien Paganini, Pierre Pontarotti

**Affiliations:** ^1^ XEGEN, Gemenos, France; ^2^ Aix Marseille Université, IRD, APHM, MEPHI, IHU Méditerranée Infection, Marseille, France; ^3^ SNC5039 CNRS, Marseille, France

**Keywords:** convergent evolution, kin recognition, vegetative incompatibility, self incompatibility, somatic diversification, adaptive immune system evolution

## Abstract

Highly polymorphic loci evolved many times over the history of species. These polymorphic loci are involved in three types of functions: kind recognition, self-incompatibility, and the jawed vertebrate adaptive immune system (AIS). In the first part of this perspective, we reanalyzed and described some cases of polymorphic loci reported in the literature. There is a convergent evolution within each functional category and between functional categories, suggesting that the emergence of these self/non-self recognition loci has occurred multiple times throughout the evolutionary history. Most of the highly polymorphic loci are coding for proteins that have a homophilic interaction or heterophilic interaction between linked loci, leading to self or non-self-recognition. The highly polymorphic MHCs, which are involved in the AIS have a different functional mechanism, as they interact through presented self or non-self-peptides with T cell receptors, whose diversity is generated by somatic recombination. Here we propose a mechanism called “the capacity of recognition competition mechanism” that might contribute to the evolution of MHC polymorphism. We propose that the published cases corresponding to these three biological categories represent a small part of what can be found throughout the tree of life, and that similar mechanisms will be found many times, including the one where polymorphic loci interact with somatically generated loci.

## Preamble

**Figure 1:**
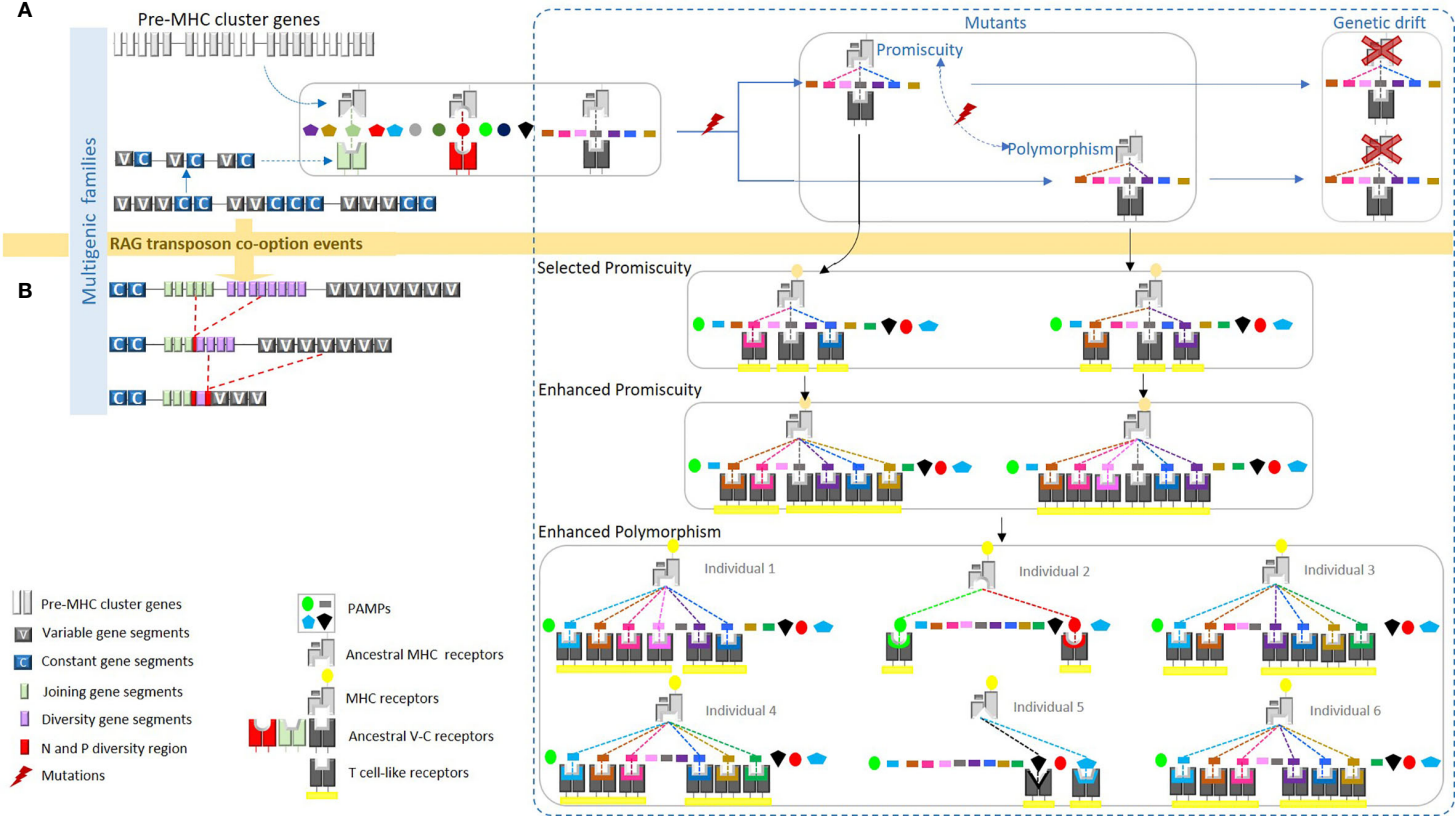
We proposed a hypothetical model whereby the ancestral MHC-like molecule bound certain pathogen-associated molecular patterns (PAMPs), presenting them to ancestral TCR-like molecules. **(A)** The ancestral MHC-like molecule may have been limited to just a few pathogens and each ancestral TCR-like molecule might have recognized only a particular class of PAMP bound to the ancestral MHC-like molecule. A mutation in the ancestral MHC-like molecule may have allowed the binding of a new PAMP, but this new combination could not be recognized by the ancestral TCR-like molecule. As a result, the new MHC-like molecule would be lost by genetic drift. **(B)** The integration of the recombination-activating gene (RAG) transposon into an ancestral TCR-like gene may have led to a significantly increased probability of recognition by these original, non-diverse TCRs. Thus, mutations in the ancestral MHC-like molecule may have led to a conformational ability to bind new PAMPs; consequently, mutated MHC molecules could have been recognized by diverse TCRs thereafter, becoming evolutionarily selected via three mechanisms: peptide-binding promiscuity, allelic polymorphism, and expansion into multigene families. The expanded ability of this ancient MHC/TCR system to recognize new PAMPs would presumably allow peptides to be bound, presented, and recognized during an immune response. We propose in the present article that this phenomenon could be universal. This figure has been reprinted with permission from Tsakou-Ngouafo et al. (1).

We develop this work mainly to determine whether MHC-like systems could be found in phyla other than vertebrates. We define an MHC-like system as consisting of a polymorphic presenting molecule (the MHC) that recognizes a small molecule and interacts with a receptor capable of somatic diversification. The possibility that MHC-like could be polymorphic is based on our earlier proposition: the MHC polymorphism is due to the limited binding properties of the MHC binding pocket, compared to the capacity of recognition of the T cell receptor [(1), see **Figure 1A**].

We developed here this proposal and named it “the capacity of recognition competition mechanism” (CRCM) (see below). We further propose that MHC-like systems evolved in other phyla than that of the jawed vertebrates.

This proposal is inspired by two kinds of observations:

The first kind of observation leads to the hypothesis that diversity, driven by somatic recombination, evolved many times during species evolution. Indeed the recombination activation gene (RAG), which is involved in the somatic TCR rearrangement, is a domesticated cut and paste transposon (1, 2) belonging to the DDE transposon family. It has to be noted that the number of DDE transposons found in nature is huge (2) and that DDE transposons have been co-opted several times as site specific recombinase.

The second kind of observation is that tripartite interaction is found in other cases that MHC plus peptide and TCR interact. This has been described in the following case: BTN3A1 recognizes, *via* an internal domain, a pathogen metabolite leading to a modification of the conformation of the extracellular domain leading to the activation of the V*γ*9V*δ*2 TCR (3).

Our article is organized as follows: we first describe the different biological categories in which polymorphism loci are involved: 1) kind recognition, 2) self-incompatibility (inter-individual recognition, 3) the MHC system involved in the jawed vertebrate adaptive immunity.

We will describe that recognition primarily occurs through the interaction of polymorphic ligands and polymorphic receptors and at least in one case of interaction between receptors driven by a somatic mutation and polymorphic ligands. We will then present the hypothesis that predicts the MHC-like systems exist in phyla other than jawed vertebrates.

In this article, we discuss the diversity of life using examples of different clades of species. In order to better follow the text, we advise readers to refer to the Tree of Life [(4) http://tolweb.org/tree/].

## Kind Recognition

Kin recognition is the body’s ability to distinguish between close genetic individual (kin) and less genetically related individual (non-kin). This capacity is found throughout the diversity of life. Different mechanisms have been implemented to enforce cooperation between cells. The interaction can be helpful or harmful (5) rewarding kinship and/or penalizing non-kinship. It is important to note that, the interaction can be limited at certain loci, a process named “kind recognition”. Much discrimination appears to be achieved through kind recognition rather than kin-recognition. The genes involved in kind-recognition are named “green beard” genes. Many of the described Greenbeard genes are polymorphic. The recognition between the ligand and the receptor can correspond to homotypic or heterotypic interaction. In the case of heterotypic interaction, the genes coding for the interaction protein are linked in the genome. Recognition could be self-recognition, and this has led to cooperation, non-self-recognition; in this case it will be harmful and missing self-harmful.

### Bacteria

Kind recognition has been well described for two bacterial species: *Proteus Mirabilis* and *Myxococcus Xanthus.*

#### Proteus mirabilis

*Proteus mirabilis* is capable of forming swarming colonies. Genetically different colonies do not interact, while those of genetically identical populations merge (6). Id proteins are important for the recognition mechanism. Indeed, IdsD and IdsE encode identity information for each colony. Heterotypic complex is formed between polymorphic *IdsD* and *IdsE* (7) belonging to the same operon and therefore are strongly genetically linked. They bind also *in vitro* in an allele-restrictive manner. Saak and Gibbs (6) demonstrated that the non-interaction between *IdsD* and *IdsE* does not cause cell death. The non-interaction leads to restricting the expansion radius of the swarming colony. Therefore, *P. mirabilis* communicates Ids between neighboring cells for a helpful interaction (6).

#### Myxobacteria

At least two systems are involved in the recognition; they operate in a subsequent manner. The first step involves the TraA/TraB receptor-ligand binding. The second step is a verification step; a polymorphic toxin is delivered to the recognized cell. Genetically identical cells will express a related immunity factor and survive, while a cell that is not genetically identical will lack immunity and die (8).

The first step involves receptor–ligand binding (TraA/TraB), Myxobacterial cells can exchange their outer membrane (OME) lipoproteins OME. TraA and TraB are cell surface proteins that must be present on both interacting cells. TraA/TraB catalyze the fusion of the OME; TraA is the receptor involved in recognition. Recognition occurs if the partnering cells have identical or very similar TraA receptors. *TraA* is polymorphic, with hundreds of different alleles (9) and here, the self-recognition led to a cooperative interaction (helpful) and the missing self is therefore harmful.

During the verification step, polymorphic toxins are delivered to the recognized cell. If the two cells are from the same strain, the recognized cell will express a cognate immunity factor and survive, whereas a cell that is not from the same strain will not possess the cognate receptor and therefore will be killed (10). This mechanism, as described for other bacteria, corresponds to a toxin–antitoxin system (11). The polymorphic toxins are delivered between cells in close contact (SitA toxins). Each toxin is associated with an antitoxin, which specifically neutralizes toxicity in the producing cell and in the cells from the same strain. As expected, the genes that code for the toxin and the antitoxin are genetically linked and are coded by the same operon. Here, the missing self is therefore harmful.

### Eukaryotes

Several eukaryotes are able of kind recognition; some of these mechanisms are known at the molecular level. Several examples are found in unicellular eukaryotes.

#### Dictyostelium discoideum

*Dictyostelium discoideum* have a unicellular life phase, but they aggregate upon starvation and form fruiting bodies with viable spores and dead stalk cells. The cell aggregation can lead to chimerism. *Dictyostelium* avoids chimerism by preferential cooperation with kin. This recognition (that participates in cell-adhesion and signaling) involves the highly polymorphic TgrB1 and TgrC1 receptors that are encoded by two adjacent genes. A pair of *TgrB1* and *TgrC1* alleles from the same haplotype is necessary and sufficient for the self-recognition, which is mediated by differential cell–cell adhesion. TgrB1 and TgrC1 receptors mediate this adhesion through direct binding. In fact (12–14), here, the self-recognition led to a cooperative interaction (helpful).

##### Fungi (here the non-kin recognition is called Vegetative incompatibility)

The systems work at two different levels: one acts post-fusion and the other pre-fusion.

##### Self-/Non-Self-Discriminations That Act Post-Fusion

Non-self-harmful heterotypic linked loci or homotypic polymorphisms are distributed between several loci; the different loci have the same functional properties. Here, the mechanism is different than above since the interaction occurs in the non-self-manner in ascomycetes, and basidiomycetes allorecognition can result in vegetative incompatibility. This is done *via* programmed cell death that occurs after fusion of cells from different strains. The recognition involves the *het* loci, with co-expression of any combination of incompatible alleles resulting in vegetative incompatibility. The Het code for a recognition domain and a cell death inducing domain, these domains can be found on the same protein or on different proteins. The recognition domain includes at least one polymorphic site, which defines allele specificity. Polymorphism at *het* loci can also involve several genes maintained in tight linkage disequilibrium, which prevents recombination. This is important in the case of heterotypic interaction (15–18).

Recognition is driven by several loci with two or three alleles of high specificity supported by a low polymorphism and several paralogous loci. For example, 11 loci are functional in allorecognition in the ascomycete *Neurospora crassa* (19).

Here, the loci are not linked; the explanation is that in natural population, events of out-crossing, which eventually lead to the formation of self-incompatible meiotic, descendants may occur very rarely. These fungi strains are pseudo-homothallic; they have two mating-types, but spores are binucleated and almost always carry nucleic of the two sexual types. So in the end, the strains can be self-fertile and can do very little outcrossing.

##### Self-/Non-Self-Discriminations That Act Pre-Fusion 

In ascomycete fungi, several loci are involved in self-/non-self-discriminations that act before the fusion step during chemotropic interactions (20). This has been well studied in *Neurospora crassa*. Recognition and chemotropic interactions between genetically identical germinated asexual spores (germlings) require oscillation of a signal transduction protein complex. Different groups of communication can be identified, in which germlings within one communication group are able to interact, while germlings from different groups of communication groups avoid each other.

Three highly polymorphic genes (*doc-1*, *doc-2*, and *doc-3*) found in linkage disequilibrium are involved in the communication group phenotype. The exchange of *doc-1* and *doc-2* alleles from different communication group strains was necessary and sufficient to define communication group identity. The ligands are not known. The deletion of *doc-1* and *doc-2* allows self-communication and chemotrophic interactions between non-communication groups. It is possible *doc-1* and *doc-2* repress a functional cascade if non-genetically identical germlings are close. Therefore, the cascade is activated when the self is missing.

The question is whether the ligand gene is close to the doc loci, or do we have some kind of education (change at the somatic level)? This is an important point to raise and will be discussed in the hypothesis section of the article.

#### Metazoan

Kind recognitions have been described in several clades of colonial metazoan (and likely to be present in all the colonial metazoan) desmoponges, cnidarians, ascidians, bryozoans, see for review Grosberg (21); most of the cases have been evidenced *via* artificial tests, such as grafting. The cases characterized *in vivo* are described below.

##### Cnidarian: Hydractinia

*Hydractinia* polyps, which are asexual, can join and form colonies; the junction is done *via* vascular-type canals. These vascular-type canals allow the stem cells of one individual to migrate into the vasculature of the fusion partner. Three outcomes can be evidenced in transplantation assays: fusion, transitory fusion, and rejection. This is controlled by highly polymorphic loci *Alr1* and *Alr2* that encode self-ligand protein. If colonies share at least one allele at both genes, they fuse. If an allele is shared in only one of the two genes, the colonial fusion is transient. The rejection occurs when the two individuals have different alleles for the two genes.

Hundreds of *Alr2* alleles have been described (22, 23) in natural population of *Hydractinia* (22, 23), while *Alr1* are expected to be similarly polymorphic (22).

##### Urochordate Botryllus

*Botryllus schlosseri* colonies’ contact leads to fusion or rejection. In fusion, each colony’s tunic border dissolves at the contact zone; the opposing matrices and ampullae fuse, leading to a continuous circulation system. In rejection, a cell mediated inflammatory reaction prevents vascular continuity. The cells involved in the cytotoxic reaction are the morula cells [see for review (24)]. The fusion or rejection outcome is controlled by the histocompatibility locus *FUHC* fusion/histocompatibility (25). Fusion will occur between two colonies if they share one or both haplotypes of the *Fuhc* locus, while rejection will occur if two colonies share no alleles.

Given the rarity of fusion observed between randomly paired colonies, *FUHC* has been inferred to be highly polymorphic. Several polymorphic loci are found in the *FUHC* cluster, one of them, *BHF*, seems to play a crucial role in the allorecognition (26, 27).

Voskoboynik and colleagues (26) showed that polymorphisms on the BHF locus agree with the fusion rejection pattern, and other polymorphic loci found in the vicinity of BHF have poor fusion/rejection predictability. These outcomes have been viewed with some skepticism (24). Indeed, the same analysis suggested that *cfuhcsec* and *cfuhctm*, *hsp40-l*, which are separated respectively 227 bp and 8 kb each and are in strong linkage disequilibrium, had different predictive abilities. However, an interesting observation published in 2018 (27) reported that *B. schlosseri* Morula cells do not express BHF protein on the cell membrane. This suggests the existence of an inhibitory receptor on the morula cytotoxic cells that can recognize self-BHF. It is important to note that one of the genes that is differentially expressed by cytotoxic morula cell is *sFuHC*. Given the mechanism of inhibition of BHF and the significantly high expression of sFuHC in cytotoxic morua cells, it is likely that FuHC acts as a BHF inhibitory recognition receptor.

## Self-Incompatibility in Sexual Reproduction and Mating System

In animals and plants, the sexual reproduction is due to the fusion of two gamete types of different sizes (anisogamy). In both phyla, self-incompatibility has been developed for hermaphrodites species in order to avoid self-fertilization and thus polymorphism depression. The self-incompatibility can be determined at the organism phenotypic level or at the gametic genetic level. We will focus here on the studies investigating the gametic level.

In addition to plants and animals, most of eukaryotes reproduce *via* the same gamete type isogamy. In order to avoid “polymorphism depression”, they use the mating system. The best studied case is the one of the basidiomycetes fungi.

### Mating in Basidiomycetes Fungi

In Basidiomycetes fungi, sexual reproduction is often driven by two independent sets of mating type (MAT) specific genes that control different stages of the sexual cycle. This is supported by two unlinked loci: the *HD* and the *P/R* loci. The *H/D* loci encode transcriptional regulators. The encoded proteins are HD1 and HD2. They correspond to two classes of homeodomain transcription that form heterodimers regulating post-mating behavior (28, 29). The HD1 interacts with the HD2 from a different allelic form than the HD2 self and *vice versa*. The number of *HD1–HD2* alleles, for example in *Coprinopsis Cinera*, is estimated at 160. *PR* locus encodes one pre-mating lipopeptide pheromones and their receptors (P/Rs), which mediate recognition of mating partners and cell fusion; the pheromone will interact with the receptor of a different allelic form in a given allelic group. The number of (*P/R*) loci in the case of *Coprinopsis Cinera* has been estimated at 79.

### Self-Incompatibility in Plants and Animal

In plants and metazoan, biological mechanisms exist to avoid inbreeding for the hermaphrodite; self-incompatibility can be found at the morphological (structural barrier) or genotypic level.

Two different systems have been set up during the evolutionary history of hermaphrodites: helpful non-self-recognition and harmful self-recognition.

#### Self-Incompatibility in Plant

Self-incompatibility is described in approximately 40% of flowering plant species and in at least 100 families. The self-incompatibility can be found at the morphological level (structural barrier) or at the genotypic level. At the genetic level, self-incompatibility system evolved at least three times during the eudicot evolution and at least once in monocots (30).

##### Non-Self-Recognition Protection: The Most Well Described Cases Is the One of Solanaceae

###### SRNASE mediated gametophytic self-incompatibility

The SRNASE mediated gametophytic self-incompatibility occurred after the monocot–dicot split, but before the rosids asterids split. It was replaced many times by polymorphic incompatibility, phenotypic incompatibility in most of the cases and has been also replaced at the molecular level in the case of brassicaceae by the sp11 SRK system and PrsS/PrpS in *papaveraceae* (sister group of the rosids asterids group).

The male and female genes are linked. The female S determinant is an S-RNAse, the male S determinant encodes an F-box protein (SLF), which recognizes and detoxifies the female-determining S-RNases from another haplotype. The SLF forms multigene families (formed of several paralogs). Each paralogous SLF protein specifically interacts with one or more S-RNAse proteins of other S-haplotypes. High polymorphism and sequence divergence of the SRNase genes and conservation of a high sequence of *SLF* genes from different haplotypes are found (30–32).

The explanation of the processes is that the *SLF*, the paralog recognizing the self S-RNAse, is counter-selected as this would result in polymorphism depression leading to decreased fitness (33).

##### Self-Incompatibility via Self-Recognition in Plant

Here, the recognition of self prevents fertilization. This results in tight coevolution between male and female determining components in a single haplotype. If the two loci are separated, fertilization can occur and cause inbreeding depression, so the two loci must co-evolve.

These self-histocompatibility mechanisms have been very well studied in two model groups: *Brassicaceae* and *Papaveraceae.*

###### Brassicaceae

The S-locus in the *Brassicaceae* carries two tightly linked and highly polymorphic S-determinant genes. The S-Locus protein 11 (SP11) or S-Locus cysteine rich protein (SCR) is the male S-determinant: SP11 is a secreted peptide located in the pollen coat. The S-locus Receptor Kinase (SRK) is the female S-determinant. SRK corresponds to a transmembrane protein belonging to the ser/thr receptor kinase family. SRK is located on the plasma membrane of the stigma papilla cells. Direct and specific molecular interaction between SP11 and SRK from the same S-Haplotype induces the incompatibility response in the stigma leading to self-pollen rejection.

###### Papaveracea

Male and female S determinants carry two closely related and highly polymorphic genes. The female S determinant: *PrsS* encodes a small protein secreted by papilla cells of the stigma. The male S determinant: *PrpS* encodes a small protein with several predicted transmembrane folds. The system is not as well understood as that in the case of *brassicaceae*. However, the specific interaction between PrsS and PrpS from the same S-haplotype results in self-rejection of the self (same S-haplotype); specific PrsS binding to its cognate PrpS on pollen plasma membrane elicits Ca2+ influx in the pollen tube, which triggers SI responses, leading to programmed cell death.

#### Self-Incompatibility in Animals

Self-incompatibility has been described for many tunicates and cnidarians. However, the mechanism is only known for the ascidian *Ciona Intestinalis* (34, 35). The SI system in *Ciona intestinalis* is similar to those of *Papaveracea* and *Brassicaceae*. The products of the sperm-side and egg-side multiallelic SI genes, which are closely related and highly polymorphic, appear to be responsible for the Self-incompatibility, as revealed by genetic analysis. The male-side *S*-determinant located in sperm head surface in animals and female-side *S*-determinant located in the vitelline coat of animal eggs recognize each other. When it is recognized as the same haplotype allele, fertilization is blocked.

Self-incompatibility can be found in other hermaphrodite animals, such as coral, but the self-incompatibility system does not seem to be driven by molecular mechanism (36). More investigations have to be carried out for animals. Nonetheless, it is likely that similar molecular systems will be found.

## The MHC System

Two characteristics are specific to the MHC: 1) the system is involved in self non-self-interaction and interacts with a receptor (a tripartite recognition system); 2) the receptor (T cell receptor) diversifies at the somatic level. In order to better understand our hypothesis, it is useful to describe cases of self–self interaction and direct recognition using somatic diversification mechanisms.

### Cell–Cell Recognition From the Same Individual, Interactions Between Loci Able to Perform Alternative Splicing and Regulation of Isoform Expression

#### Neural Development: Self-Avoidance During Neural Development Cases

During the neural development in many animals, self-avoidance ensures that a neuron’s process arborizes to evenly fill a particular spatial domain (37). At the individual neuron level, self-avoidance is driven by genes encoding cell-surface molecules (38, 39). This is done by generating thousands of different isoforms due to alternative splicing, such a as *Dscam1* in drosophila and homophilic avoidance (38), or, as in the case of vertebrates, by using a homophilic interaction between different isoforms from a multigene family (protocadherin), which are expressed in a combinatorial way; a single mismatch can infer with the combinatorial homophilic interaction (39). Consequently, the recognition of cell identity is due either to the homophilic recognition of a system capable of generating thousands of isoforms, or to a system using the combination of several possible isoforms.

#### NK System

Individual Ly49 receptors in mouse and KIRs in humans are expressed in a stochastic and independent manner on NK cells. This mechanism allows the formation of a unique ‘NK cell repertoire’ in each individual, which is characterized by the coexistence of several NK cell subsets, each expressing from zero to five receptors. The composition of the NK cell repertoire is determined by several factors. Among these latter, the MHC repertoire is an important player as it affects the composition of the final Ly49/KIR repertoire and thus contributes to NK cell education. The NK cell education results from the differential interaction between the different MHC alleles and NK receptors.

It has to be noted that the interaction between the NK receptors and MHCs could be very ancient (40) and that the NK system may therefore have played a role in the emergence of MHC polymorphism (41).

However, to date, the NK system is not well known outside of the mammalian phyla. This will be discussed later in the article.

### Direct Recognition With Self and Non-Self *via* Somatic Diversifying Selection

#### Olfactory System (Direct Interaction With Non-Self)

The odorant receptor genes (ORs) encode seven transmembrane receptor proteins that form the largest mammalian protein family. ORs are expressed monogenically and monoallelicaly in each olfactory epithelium neuron. The olfactory system is fantastically discriminating, so it uses a combinatorial receptor coding scheme to encode olfactory identities. Indeed, one OR can recognize several odorant molecules, and one given odorant is recognized by multiple ORs. However, the different odorant molecules are recognized by different combinations of ORs (42).

#### Immunoglobulin Diversity (Direct Interaction With the Self and Non-Self)

The jawed vertebrates’ adaptive immune system includes the immunoglobulin, the T-cell receptors (TCR) and the MHC. Immunoglobulin and TCR recognize antigenic determinants through the variable (V) regions, whose diversity is generated by V(D)J module recombination. The Immunoglobulin and TCR repertoire is increased thanks to the junctional diversity. This process corresponds to the addition of non-templated encoded nucleotides (N) at V(D)J junctions. Immunoglobulin directly recognized antigen, while, in the case of TCR, the recognition of the antigen involved a presentation of the antigen *via* the MHC, see after in text. The recombination-activating gene products (RAG proteins) constitute the enzymatic core of the V(D)J recombination machinery of jawed vertebrates. RAG catalyzes random assembly of variable, diverse, and joining gene segments that are present in the jawed vertebrate genome in numerous copies and together with hyper-mutation generate the enormous diversity of the assembled antibodies and T-cell receptors. The rearrangement occurs *via* repetitive elements at the edge of the rearranging segments, having a conserved heptamer and nonamer sequence separated by a space of either 12 or 23 base pairs: the recombination signal sequences or RSS [see for review Teng (43) and references]. The VDJ recombination mechanism is similar to the DDE transposon. Indeed, the DDE transposons encode for a transposase coding gene flanked by two Terminal Inverted Repeats (TIRs). To achieve transposition, the transposase recognizes these TIRs to perform the excision of the transposon which is then, or in concert with the excision, reinserted into a new genomic location. A DDE transposon (DNA cut and paste transpososon) named the RAG transposon, which is homolog to the RAG VDJ, has been described in non-vertebrate species (see for review 1). A likely hypothesis is that that the RAG DDE transposon invaded in the jawed vertebrate ancestor an IG-Like gene eventually resulting in the antibody/TCR rearrangement (1, 44).

A different recombination system is used in birds and some mammals to generate the immunoglobulin diversity. Indeed, only one pair of functional V and J segments are found for both the Ig light and heavy chain loci, and several pseudo V coding segments are found upstream the functional V segments in genes coding light and heavy chains. These pseudo-V segments are used as template for gene conversion to diversify the single functional V segment. To launch the diversification process, a break done by the Activation-induced deaminase (AID) within the single functional V segment is required [see for example (45)].

#### The Agnathe VLR System*/*Direct and Maybe Tripartite Recognition System

In cyclostomes (agnathe vertebrates), the variable lymphocyte receptors (VLRs), the VLR system involved in adaptive immunity includes a germline VLR that does not code for functional protein, but encodes instead the only portion of the amino and carboxyl termini of the mature VLRs. The sequences encoding those portions are separated by non-coding intervening regions. In lymphocytes, the germline VLRs are assembled by somatic DNA rearrangement into a mature VLR that encodes the functional receptor *via* the insertion of LRR cassette that flanks the germline VLR. The germline VLR is broken by the AID-APOBEC enzyme at the intervening sequences between the C terminal and N terminal portion of the VLR genes. Then, gene conversion starts, thanks to sequence identity between the intervening sequence and sequences surrounding the LRR cassettes (46). The VLR could be secreted (expressed in agnathes B cell like): VLRB or transmembrane protein VLRA and VLRC (which are expressed in *αβ*T cell like *γδ* T cell like). It is not known whether VLRA and VLRC interact with an MHC analogous structure.

#### MHC/T-Cell Receptor Somatic Diversity and Self Non-Self-Recognition: The Capacity of Recognition Competition Mechanism Hypothesis

The central interaction in cell-mediated adaptive immunity lies between the *αβ*T cell antigen receptor (*αβ* TCR) and MHC class I and MHC class II antigen-presenting molecule loaded with peptides. MHCs are highly polymorphic genes: more than 6,000 MHC allomorphs have been described so far (47). The polymorphism is localized around the peptide-binding cleft of the MHC protein. The different MHC allomorphs have distinct peptide-binding preferences that are determined by anchor residues that reside within MHC pockets (48). MHC allomorphs can present several thousand peptides (49) with more or less overlaps between loci. The combination of MHC plus peptide is recognized by T cell receptors. The generation of the T cell receptor diversity occurs during T cell ontogeny in the thymus. The size of the TCR repertoire has been estimated to be around 2 × 107 (50). Further, TCRs recognize multiple peptide/MHC complexes (TCR cross-reactivity or polyspecificity) (51–53).

Therefore, MHC proteins have a limited capacity of presenting peptides compared to the capacity of the TCR to recognize different sets of MHC plus peptides. This could explain MHC polymorphism. Indeed, new variations given to a new peptide interaction that would be recognized by a T cell receptor could not be fixed in an individual as this would result in the loss of the binding property of another peptide from a given pathogen present in the environment. Therefore, the mutation will be counter-selected. However, the fixation can occur in an individual living in an environment where the pathogen is absent, leading to polymorphism. We name this mechanism: The capacity of recognition competition mechanism.

We first proposed this mechanism to explain the origin of the MHC polymorphism in the context of the origin of the adaptive system of jawed vertebrates [**Figure 1** and (1)]. We proposed, before the origin of the jawed vertebrate adaptive immune system, that the pre-MHC system was able to recognize some Pathogen-associated molecular patterns (PAMPs) and present them to receptors able to recognize the pre-MHC plus the PAMPs. The evolution of the system was low and likely limited to some pathogens. Indeed, even if the receptors (T cell like receptor) could be encoded by large multigenic families, and this was likely (1), they could recognize only a few couples pre-MHC/PAMPs. Therefore, even though pre-MHC could recognize a new antigen, thanks to a new mutation, as this new couple will not be recognized by the T cell like receptor, substitutions will not be selected and eventually lost (maybe the new MHC variant will lose the possibility to interact with another pathogen).

T cell like receptors could have increased their diversification, thanks to the integration of the RAG transposon in the module of recognition of the ancestor of the T cell-like receptor. This event led to an increase in the recognition possibilities (1, 44), as many receptors could be present, thanks to rearrangement. Therefore, mutations in the pre-MHC leading to a recognition of new pathogens could be positively selected. Of course, selection of polymorphic MHC could be also due to other interactions, such as the one with the NK receptor that can enhance the MHC polymorphism, but this is not incompatible with the capacity of recognition competition mechanism.

In the next paragraph, we will propose that receptors that encoded highly polymorphic loci interacting with receptors generated by somatic mutations could be present in many systems involved in the adaptive recognition processes.

## Proposition: The Tip of the Iceberg

Our analysis only describes a very small part of the cases present in nature. It is likely that the receptor for interacting polymorphic ligands will be found several times in the case of loci involved in the kin recognition and self-incompatibility.

Many cases of kind and self-incompatibility have been reported in the literature, but the molecular mechanism remains unknown.

For kind recognition, many examples are found in: protists (54), a colonial vertebrate (55), and bacteria (9).

There are many cases of self-incompatibility in plants and animals [see for example (36)] and it is likely that species with several types of mating are found in eukaryotes, because most of them are isogamous eukaryotes (56). We can predict that, in these cases, polymorphic interacting loci will be found. They will be easy to identity *via* population genomic analysis, as shown very nicely by Zhao et al. (19).

What about the MHC-like systems? We mainly carried out these analyses in order to assess whether MHC-like systems could be found in phyla other than vertebrates. We defined an MHC-like system as a system that consists in presenting molecules encoded by polymorphic loci that recognize small molecules and interact with receptors generated *via* somatic diversification. The possibility that MHC-like could be polymorphic is based on our earlier proposition that the MHC polymorphism is due to the limited binding properties of the MHC binding pocket (1) (see **Figure 1A**) compared to the capacity of recognition of the T cell receptor (almost unlimited). A phenomenon that we called “the capacity of recognition competition mechanism” (see above).

We propose that other somatically diversifying systems could have driven the emergence of polymorphic loci *via* non-self-abiotic or biotic component. This is because mechanisms of the generation of somatic diversity and tripartite interactions evolved many times during life history. Indeed, rearranging processes involved in the gene somatic diversification evolved in a convergent manner *via* the convergent use of gene conversion (VLR) or *via* the use of site specific recombinase systems (RAG) [(1) see **Figure 1B**]. As mentioned earlier, RAG is a domesticated RAG transposon. The main difference between the VDJ RAG and the RAG transposon is the fact that the excised fragments in the case of the VDJ recombination lost the properties to insert new genomic location by losing the integrase activity of the transposase (1). This evolutionary functional shift did not require dramatic changes. This must be related to the fact that many DDE transposons have been recruited as site-specific recombination system several times during evolution (2). As the number of DDE transposons found in nature is huge (57), we propose that some of them could have been recruited as site specific recombinase involved in the generation of high somatic diversity and, therefore, in the capacity of recognition competition mechanism leading to polymorphic counter receptors.

The MHC system is not the only one capable of a tripartite interaction. Indeed, V*γ*9V*δ*2 T-cells detect tumor cells and microbial infections through the recognition of phosphoantigens (pAgs). The recognition is mediated by a member of the butrophylin family: butyrophilin 3A1 (BTN3A1). The intracellular domain of BTN3A 1: B30.2 binds pAg through a positively charged surface pocket inducing a modification of the conformation of the membrane butyrophilin BTN3A1. This conformation shift is recognized by the V*γ*9V*δ*2 TCR (58) and drives T cell activation. In that case, *BTN3A1* is not polymorphic. However, this shows that tripartite interaction involving a presenting molecule that interacts with a self or non self molecule forming a complex recognized by a receptor evolved in a convergent manner.

## Conclusion and Perspectives

In conclusion, we hypothesize that other MHC-like systems will be found in other living organisms. In the next chapter we propose a strategy to search for them and discuss their possible function.

### How to Find MHC-Like Systems?

The strategy combines two approaches: the search for polymorphic loci and for rearranging loci. If both loci are found for the same organism, it could be a MHC/TCR-like complex.

**Search for polymorphic loci:** To evidence such loci, the strategy to used is the following: to have a genome of reference and many sequenced genomes from the same species (no need to finish sequences); map the polymorphic sites and look for the most polymorphic loci (they can be several); they could be the candidate, see (19, 59, 60).

**Search for somatic diversification:** Somatic diversification can occur *via* different mechanism rearrangements, expression of different combinations of paralogs, like the protocadherin example, but we will need a lot of paralogs. The same can be said for alternative splicing. The rearrangement strategy seems to be a more efficient system to generate diversity. Rearrangement can occur *via* different mechanisms (site directed recombination, gene conversion…). So far, many simple programmed rearrangements (rearranging two loci) have been described [see for a complete review (1, 2)]. Rearrangement should occur between several sets of paralogous genes with a combinational joining (a set of germ line linked paralog gene segments), able of combinational joining with segments of another set of linked paralog gene segments to create a functional loci (able to code for a protein) with the addition of diversity. This could be driven *via* gene conversion, where a module copy in a *bona fide* gene is replaced by a module. Except if the replacing modules are huge, we do not expect a lot of diversity from this process. However, the process that occurs *via* a domesticated DDE transposon is expected to increase diversity *via* the p diversity and n diversity. The p diversity is due to the way the domesticated DDE transposon cuts at the recognition sequence. We know that RAG and some families of DDE transposons [see for review (1)] are able to create hairpin when they excise their hairpin and are then cut in an asymmetric way *via* the nuclease. Artemis will provide later palindromic diversity during the junction. Further, some nucleotides can be deleted, so domesticated DDE transposon able to generate hairpin could be the champion of the generation of diversity. The experimental *in silico* design would be as follows: look for a similar organization than the IG and TCR locus: we will look, for example, for a gene fragment named A and a gene fragment named B that are linked on the same transcript, with the following organization at the genomic level: A found in multi-copies, with each copy close to each other, followed by fragment B with the same organization as A. We will evidence the RSS bordering A and B by comparing the sequence flanking A and B with the TIR from the different transposon families. The family of transposons where the RSS comes from will therefore be identified, and this will allow the domesticated transposase to be evidenced. The genome of the cell or tissue where the domesticated transposase is expressed will be sequenced and compared to the germline genome.

### What Could Be the Function of MHC-Like System?

Intuitively, we could think that the MHC-like system could be involved in other possible cases of adaptive immunity or in the recognition of very diverse elements present in the environment, such as odors that could influence our comportment. However, it could also be involved in kin recognition as well. Indeed, vertebrate MHC is involved—as its name says—in graft rejection in case of histocompatibility. The graft rejection is due in fact to the positive and negative selection of the TCR, which, depending on the MHC allotype (and also on the minor histocompatibility antigens), will result in two different repertoires, and some TCRs from the individual A reacts with the MHC of an individual B (if the allotype is different). Even if this is artificial (graft does not occur in nature, at least in vertebrate), it is still a possibility that h colonial species could use a MHC-like system.

## Data Availability Statement

The original contributions presented in the study are included in the article/supplementary material. further inquiries can be directed to the corresponding authors.

## Author Contributions

Analysis and writing the manuscript: JP and PP. All authors contributed to the article and approved the submitted version.

## Funding

This research was funded by the French Government under the “Investissements D’avenir” (Investments for the Future) programme managed by the Agence Nationale de la Recherche (ANR, fr: National Agency for Research), (reference: Méditerranée Infection 10-IAHU-03) and XEGEN R&D company funding.

## Conflict of Interest

The authors declare that the research was conducted in the absence of any commercial or financial relationships that could be construed as a potential conflict of interest.
